# Clinical and pathological features of immune-mediated necrotising myopathies in a single-centre muscle biopsy cohort

**DOI:** 10.1186/s12891-022-05372-z

**Published:** 2022-05-06

**Authors:** Hongxia Yang, Xiaolan Tian, Lining Zhang, Wenli Li, Qingyan Liu, Wei Jiang, Qinglin Peng, Guochun Wang, Xin Lu

**Affiliations:** 1 China-Japan Friendship School of Clinical Medicine, Peking University, Beijing, 100029 China; 2 Department of Rheumatology, China-Japan Friendship Hospital, Yinghua East Road, Chaoyang District, Beijing, 100029 China

**Keywords:** Muscle biopsy, Idiopathic inflammatory myopathies, Immune-mediated necrotising myopathy, Pathological features

## Abstract

**Objective:**

Immune-mediated necrotising myopathy (IMNM) is a subset of idiopathic inflammatory myopathies (IIM) characterized by significantly elevated creatine kinase level, muscle weakness and predominant muscle fibre necrosis in muscle biopsy. This study aimed to investigate the clinical and pathological characteristics of patients with IMNM in a single-centre muscle biopsy cohort.

**Methods:**

A total of 860 patients who had muscle biopsy reports in our centre from May 2008 to December 2017 were enrolled in this study. IMNM was diagnosed according to the 2018 European Neuromuscular Centre (ENMC) clinicopathological diagnostic criteria for IMNM.

**Results:**

The muscle biopsy cohort consisted of 531 patients with IIM (61.7%), 253 patients with non-IIM (29.4%), and 76 undiagnosed patients (8.8%). IIM cases were classified as IMNM (68[7.9%]), dermatomyositis (346[40.2%]), anti-synthetase syndrome (82[9.5%]), polymyositis (32[3.7%]), and sporadic inclusion body myositis (3[0.3%]). Limb girdle muscular dystrophy (LGMD) 2B and lipid storage myopathy (LSM) are the two most common non-IIM disorders in our muscle biopsy cohort. IMNM patients had a higher onset age (41.57 ± 14.45 vs 21.66 ± 7.86 and 24.56 ± 10.78, *p* < .0001), shorter duration (21.79 ± 26.01 vs 66.69 ± 67.67 and 24.56 ± 10.78, *p* < .0001), and more frequent dysphagia (35.3% vs. 3.4 and 6.3%, *p* = .001) than LGMD 2B and LSM patients. Muscle biopsy from IMNM showed more frequent muscle fibre necrosis (95.6% vs 72.4 and 56.3%, *p* < .0001), overexpression of major histocompatibility complex-I on sarcolemma (83.8% vs 37.9 and 12.9%, *p* < .0001), and CD4^+^ T cell endomysia infiltration (89.7% vs 53.6 and 50%, *p* < .0001) compared with those from LGMD 2B and LSM patients.

**Conclusions:**

It is easy to distinguish IMNM from other IIM subtypes according to clinical symptoms and myositis specific antibodies profiles. However, distinguishing IMNM from disorders clinically similar to non-IIM needs combined clinical, serological and pathological features.

**Supplementary Information:**

The online version contains supplementary material available at 10.1186/s12891-022-05372-z.

## Background

Idiopathic inflammatory myopathies (IIM) are a group of heterogeneous autoimmune diseases characterised by inflammatory infiltration of the skeletal muscle, elevated creatine kinase (CK) levels, and muscle weakness [1, 2]. Conventionally, early IIM is classified into dermatomyositis (DM) and polymyositis (PM), based on the presence or absence of a rash [3, 4]. However, subsequent studies have found that the pathological characteristics of PM and DM are completely different. The invasion of non-necrotic muscle fibres by cytotoxic CD8^+^ T cells and upregulation of major histocompatibility complex (MHC)-I on the sarcolemma are key pathological diagnostic features of PM [5]. However, more studies have found that typical pathological characteristics of the CD8^+^ T/MHC-I are not common in PM and that PM has been overdiagnosed [6]. Therefore, the European Neuromuscular Centre (ENMC) proposed a new subclass of IIM with pathological manifestations of myocyte necrosis and less inflammation, called immune-mediated necrotising myopathy (IMNM) in 2004 [7]. IMNM diagnostic criteria were revised by the ENMC in 2017, and myositis-specific antibody (MSA) profiles were considered in the IMNM criteria. Thus, patients with anti-signal recognition particle (SRP) or anti-3-hydroxy-3-methylglutaryl-coenzyme A reductase (HMGCR) antibodies can be diagnosed with IMNM, although IMNM cannot be excluded in seronegative patients [8]. Therefore, seronegative IMNM patients are clinically easily confused with PM patients. Other subsets of IIM include DM, anti-synthetase syndrome (ASS), and sporadic inclusion body myositis (sIBM), which have distinguished clinical features with or without specific MSA and are relatively easy to distinguish from IMNM [1, 9].

Additionally, it is difficult to distinguish IMNM from other myopathies, such as muscular dystrophy and congenital myopathy. Muscular dystrophies and metabolic myopathy represent a large group of inherited conditions that may be confused with autoimmune myopathy [10, 11]. While there are more than a hundred different types of muscular dystrophies and metabolic myopathies, this study focused on the largest number of non-IIM, limb-girdle muscular disease (LGMD) 2B and lipid storage myopathies (LSM), which are also common inherited myopathies compared with other types in the Chinese population [12]. They are also often misdiagnosed as autoimmune myopathy, because they can present with proximal muscle weakness, elevated serum muscle enzyme levels, prominent collections of inflammatory cells in muscle biopsies, and/or no family history due to an autosomal recessive inheritance pattern [13, 14]. The difference is that LGMD 2B may involve both the shoulder and pelvic girdles and onset in late adolescence to mid-adulthood [15]. As misdiagnosis can lead to inappropriate and potentially harmful therapy, accurate diagnosis is essential. Therefore, it is meaningful to analyse the clinical and pathological characteristics of IMNM in detail, especially the differences between IMNM and similar myopathies, such as LGMD 2B and LSM.

Muscle biopsy remains a key component in the evaluation of patients with neuromuscular disorders [5]. Here, we retrospectively analysed the distribution of muscular diseases in a muscle biopsy cohort retrospectively and investigated the clinical and pathological characteristics of IMNM in a single-centre muscle biopsy cohort and analysed the differences between IMNM and its mimics.

## Methods

### Patients

This study enrolled 860 patients who underwent muscle biopsy in the Department of Rheumatology of China-Japan Friendship Hospital between May 2008 and December 2017. All recruited patients’ demographic characteristics, clinical manifestations, laboratory examination results and special test results were collected retrospectively. Muscle strength was measured by the Medical Research Council ﻿Manual Muscle Testing (MMT) Scale (grade 0–5), and severe muscle weakness was defined as a grade ≤ 3 for muscle strength [16]. All patients provided informed consent. This study was approved by the Research Review Committee and Ethical Review Committee of the China-Japan Friendship Hospital (approval number: 2019-SDZL-3).

### Classification strategies and diagnosis criteria

Enrolled patients were evaluated for the diagnosis of different muscular disorders considering clinical features, laboratory data, MSA profiles, pathological characteristics and genetic phenotype comprehensively. We first determined patients if patients were IIM according to the 2017 European League Against Rheumatism (EULAR)/American College of Rheumatology (ACR) criteria for IIM [17]. IIM patients were re-classified as DM, IMNM, ASS, PM and sIBM. The diagnosis of each subtype of IIM was used the following criteria: the 2019 ENMC criteria for DM [18], the 2018 ENMC criteria for IMNM [8], the 2011 ENMC criteria for sIBM [19], and Connors criteria for ASS [20]. Clinical amyopathic DM (CADM) was defined according to Sontheimer’s criteria, including amyopathic DM and hypomyopathic DM [21]. IIM patients who did not meet any of the above sub-category criteria were classified as PM. PM was defined as the presence of muscle weakness, elevated CK levels, no skin rash, and MSA negative, and excluding sIBM, IMNM, ASS, and DM simultaneously. Clinical symptoms and muscle pathology suggesting hereditary myopathy were further evaluated by genetic testing. The remaining cases were then reviewed for an alternate cause of muscle weakness/CK elevation. These cases were grouped into the following categories: metabolic myopathy, endocrine myopathy, asymptomatic hyperCKemia, other connective tissue diseases (CTD) accompanied with skeletal muscle symptoms, neurogenic myopathy, and myopathy induced by external factors. These categories were chosen based on published review articles on the differential diagnosis of IIM and elevated CK levels [13, 14]. The categories and diagnosis strategy for non-IIM were based on the diagnostic criteria accordingly (see Additional Table 1). Cases that could not be classified in the above categories were labelled as undiagnosed.

### Detection of MSA and myositis-associated antibodies (MAA)

Sera obtained from patients were stored at − 80 °C. MSA, including anti-SRP, anti-Jo-1, anti-PL-12, anti-PL-7, anti-EJ, anti-Mi-2, anti-MDA5, anti-TIF1-γ, anti-NXP2, and anti-SAE, as well as MAA, including anti-Ku, anti-PM-Scl 100, anti-PM-Scl 75, and anti-Ro-52, were detected by immunoblots (Euroimmun, Lübeck, Germany). Anti-HMGCR autoantibodies were tested using an enzyme-linked immunosorbent assay (Inova Diagnostics Inc., San Diego, CA, USA) according to the manufacturer’s protocol.

### Muscle MRI examination

Thigh MRI were performed at the initial diagnosis. Patients underwent whole-body coronal and thigh axial MRI scans using a Philips-Ingenia 3.0 T MRI machine (Philips Medical Systems, Best, the Netherlands), which employed an orthogonal body coil and automatic moving-bed technology. The parameters of the MRI machine were as previously described [22].

The muscle MRI results were recorded in the following aspects: inflammatory oedema, fatty infiltration, muscle atrophy, and fasciitis. Inflammatory muscular oedema was defined as increased muscle signals on the STIR images and the degree of the increased signals indicated the severity of the oedema. Muscle fatty infiltration was defined as T1W high signal caused by intramuscular abnormal fat deposition. Muscle atrophy was defined as the reduction of muscle volume [22]. Two experienced and study-blind radiologists independently reviewed all images. A third radiologist with more than 20 years of experience adjudicated disagreements in musculoskeletal imaging diagnoses.

### Muscle biopsy

Muscle biopsy specimens from all patients were obtained using open-muscle biopsy. Fresh muscle biopsy specimens were cut into 7-μm frozen sections using cryostat frozen sections (Thermo Cryotome E) and stained using haematoxylin-eosin, periodic acid-Schiff (PAS), oil red O (ORO), modified Gomori’s trichrome, NADH-tetrazolium reductase, succinate dehydrogenase, cytochrome C oxidase, and myosin ATPase. Immunohistochemistry staining for dysferlin, dystrophin, α-sarcoglycans to δ-sarcoglycans, α-dystroglycans and β-dystroglycans, MHC-I, CD4, CD8, CD20, and CD68, and membrane attack complex (MAC) was performed using the avidin-biotin-peroxidase complex method as previously described [23]. All reagents used were purchased from Abcam (Cambridge, UK).

### Genetic testing

Patients with suspected hereditary myopathy determined by clinical and pathological evidence were required to undergo genetic testing by next-generation sequencing (NGS). Genomic DNA was extracted from peripheral blood or muscle tissues using standard procedures. Proband-only targeted NGS was performed by a commercial company (MyGenostics, Inc., Beijing, China) according to the manufacturer’s instructions, using a clinical exome capture panel containing 4231 disease-causing genes. Sanger sequencing with specific primers was performed to confirm the variants detected by NGS [24].

### Statistical analyses

Statistical analysis was performed using SPSS software (version 24.0; IBM Corp., Armonk, USA). Categorical variables are expressed as percentages and absolute frequencies, and continuous features are reported as mean ± standard deviation or median (interquartile range). Comparisons among different groups were performed using Student’s t test, Mann–Whitney U test, chi-square test, or Fisher’s exact test where appropriate. If overall *p* < .05, pairwise comparisons were performed, and Bonferroni correction was used. Bonferroni-adjusted *p* < .017 was considered significantly different between pairwise groups.

## Results

### Classification and distribution of diseases in the muscle biopsy cohort

This muscle biopsy cohort consisted of 860 patients with 531 IIM patients (61.7%), 253 non-IIM patients (29.4%), and 76 undiagnosed patients (8.8%) with a total of 860. The mean age of onset was (41.32 ± 16.52) years, with disease course of (32.29 ± 53.82) months. The majority of the patients were women (M:F = 310:550). IIM cases were classified as IMNM (68 [7.9%]), DM (346 [40.2%]), ASS (82 [9.5%]), PM (32 [3.7%]), and sIBM (3 [0.3%]). In the DM group, 75 patients could be classified as CADM. According to clinical characteristics, asymptomatic hyperCKemia (47 [5.5%]), endocrine myopathy (13 [1.5%]), neurogenic myopathy (19 [2.2%]), other CTD accompanied with skeletal muscle symptoms (62 [7.2%]), and myopathy induced by external factors (25[2.9%]) (including infection, exercise, and drugs) were diagnosed. LGMD2B (29 [3.4%]) and LSM (16 [0.7%]) were the most common non-IIM aetiologies in our muscle biopsy cohort (Table 1).

**Table 1:** Classification and distribution of diseases in muscle biopsy cohort

Classification of muscular diseases	Frequency	Proportion (%)
Idiopathic inflammatory myopathy	531	61.7
Dermatomyositis	346	40.2
Clinical amyopathic dermatomyositis	75	8.7
Immune-mediated necrotising myopathy	68	7.9
Anti-synthetase syndrome	82	9.5
Polymyositis	32	3.7
Sporadic inclusion body myositis	3	0.3
Non-idiopathic inflammatory myopathy	253	29.4
Muscular Dystrophy	66	7.7
Limb grindle muscular dystrophy 2B	29	3.4
Other types muscular dystrophy	37	4.3
Metabolic myopathy	21	2.4
Lipid storage myopathy	16	0.7
Mitochondrial myopathy	3	0.3
Glycogen storage disease	2	0.2
Endocrine myopathy	13	1.5
Myopathies associated with hypothyroidism	9	1.0
Hypokalemic periodic paralysis	3	0.3
Hypophosphorus rickets	1	0.1
Neurogenic myopathy	19	2.2
Other CTD accompanied with skeletal muscle symptoms	62	7.2
Myopathy induced by external factors	25	2.9
Asymptomatic hyperCKemia	47	5.5
Undiagnosed	76	8.8
Total	860	100

*CTD* Connective tissue disease, *CK* Creatine kinase

### Clinical characteristics of IMNM in the IIM group

The IIM group included 68 IMNM (12.8%), consisting of 35 anti-SRP-positive cases (51.5%), 13 anti-HMGCR-positive cases (19.1%) and 20 seronegative patients (29.4%). DM [346 (65.2%)] was the largest subgroup of IIM. In anti-HMGCR-positive patients, two of them had a history of statin exposure. DM-specific autoantibodies were present in 61.3% of DM patients, with anti-MDA5 (70 [20.9%]), anti-TIF1-γ (57 [17.1%]), anti-Mi-2(26 [7.8%]), anti-NXP2(40[12.0%]), and anti-SAE (7 [2.1%]). ASS accounted for 15.4% in IIM, with anti-Jo-1(40 [48.8%]), anti-PL-7(22 [26.8%]), anti-PL-12(8 [9.8%]), and anti-EJ (12 [14.6%]) positive. In this muscle biopsy cohort, PM was a diagnosis of exclusion and accounted for 6.0% (32/531) in IIM group. Compared with PM, IMNM had higher prevalence of severe muscle weakness (44.1% vs 9.4%, *p* < .01), dysphagia (35.3 and 18.8%, *p* < .01), muscular atrophy (14.7% vs 0, *p* < .01), and higher CK level [2289 (894, 5505) vs 392 (52, 570), *p* < .01]. However, IMNM patients present lower frequency of fever (7.4 and 18.8%, *p* < .01), arthralgia (8.8 and 28.1%, *p* < .01) than PM patients. Only 3 patients had sIBM in our cohort, with higher onset age (55 ± 11.13 years old) and longer course of disease (70 ± 45.03 months) compared with other subtypes of IIM (Table 2).

**Table 2:** Clinical characteristics of IMNM and other types of IIM

Characteristics	IMNM(*n* = 68)	DM(*n* = 346)	ASS(*n* = 82)	PM(*n* = 32)	sIBM(*n* = 3)
Female	45 (66.2)	236 (68.2)	57 (69.5)	25 (78.1)	1 (33.3)
Age of onset	41.57 ± 15.46	44.32 ± 15.61	50.06 ± 12.12	42.75 ± 15.30	55 ± 11.13
Duration (months)	21.94 ± 25.94	23.60 ± 49.18	23.50 ± 42.35	22.08 ± 35.86	70 ± 45.03
Fever	5 (7.4)	87 (25.1)	36 (43.9)	6 (18.8)	0
Loss of weight	19 (27.9)	94 (27.2)	19 (23.2)	8 (25.0)	0
Muscle weakness	62 (91.2)	253 (73.1)	54 (65.9)	22 (68.8)	3 (100)
Severe muscle weakness	30 (44.1)	59 (17.1)	4 (4.9)	3 (9.4)	1 (33.3)
Dysphagia	24 (35.3)	94 (27.2)	14 (17.1)	6 (18.8)	0
Muscular atrophy	2 (2.9)	1 (0.3)	0	0	0
Myalgia	22 (32.4)	169 (48.8)	34 (41.5)	12 (37.5)	0
Arthralgia	6 (8.8)	123 (35.5)	40 (48.8)	9 (28.1)	0
Skin involvement	15 (22.1)	334 (96.5)	52 (63.4)	3 (9.4)	1 (33.3)
Heliotrope rash	6 (8.8)	238 (68.8)	21 (25.6)	0	0
Mechanics’ hands	1 (1.5)	125 (36.1)	25 (30.5)	0	0
Gottron’s sign	3 (4.4)	214 (61.8)	27 (32.9)	0	0
V sign	6 (8.8)	198 (57.2)	16 (19.5)	1 (3.1)	1 (33.3)
Shawl sign	5 (7.4)	143 (41.3)	14 (17.1)	1 (3.1)	0
Raynaud phenomenon	1 (1.5)	27 (7.8)	9 (11.0)	3 (9.4)	0
Interstitial lung diseases	22 (32.4)	161 (46.5)	63 (76.8)	11 (34.4)	0
Malignancies	5 (7.4)	29 (8.4)	3 (3.7)	0	0
Other connective tissue diseases	5 (7.4)	39 (11.3)	16 (19.5)	10 (31.3)	0
ALT (0-40 U/L)	117 (64,241)	38 (23,76)	32 (24,87)	43 (30,50)	–
AST (0-40 U/L)	85 (42,153)	37 (22,76)	32 (18,55)	34 (21,75)	–
LDH (100-250 IU/L)	569 (347,836)	264 (200,378)	264 (209,398)	291 (217,393)	–
CK (26-200 IU/L)	2289 (894,5505)	103 (43,520)	422 (54,1066)	333 (35,1090)	–
ANA (> 1:40)	40/66 (60.6)	198/323 (61.3)	55/78 (70.5)	19 (59.4)	0
MSA	*N* = 68	*N* = 334	*N* = 82	*N* = 26	*N* = 3
Anti-MDA5	0	70 (20.9)	0	0	0
Anti-NXP2	0	40 (12.0)	0	0	0
Anti-TIF1-γ	0	57 (17.1)	0	0	0
Anti-Mi-2	0	26 (7.8)	0	0	0
Anti-SAE	0	7 (2.1)	0	0	0
Anti-Jo-1	0	0	40 (48.8)	0	0
Anti-PL-7	0	0	22 (26.8)	0	0
Anti-PL-12	0	0	8 (9.8)	0	0
Anti-EJ	0	0	12 (14.6)	0	0
Anti-SRP	35 (51.5)	0	0	0	0
Anti-HMGCR	13 (19.1)	0	0	0	0
MSA negative	20 (29.4)	134 (40.1)	0	26 (100)	0
MAA					
Anti-Ro-52	16 (24.2)	64 (19.8)	28 (35.9)	4 (12.5)	0
Anti-Ku	1 (1.5)	0	4 (4.9)	0	0
Anti- PM/Scl	1 (1.5)	4 (1.2)	1 (1.3)	0	0
Anti-AMA-M2	6 (9.2)	11 (3.4)	1 (1.3)	3 (9.4)	0

*IIM* Idiopathic inflammatory myopathies, *IMNM* Immune-mediated necrotising myopathy, *DM* Dermatomyositis, *ASS* Anti-synthetase syndrome, *PM* Polymyositis, *sIBM* Sporadic inclusion body myositis, *ALT* Alanine aminotransferase, *AST* Aspartate aminotransferase, *LDH* Lactate dehydrogenase, *CK* Creatine kinase, *ANA* Anti-nuclear antibodies, *MSA* Myositis specific antibodies, *MAA* Myositis associated antibodies

### Clinical characteristics of IMNM compared with non-IIM

LGMD 2B and LSM are the two most common non-IIM disorders that could be definitively diagnosed in our muscle biopsy cohort, accounting for 11.5 and 6.3% of non-IIM cases, respectively. LGMD 2B and LSM patients shared similar clinical and laboratory features of muscle weakness and elevated CK levels with IMNM. However, IMNM patients had a higher onset age (41.57 ± 14.45 vs 21.66 ± 7.86 and 24.56 ± 10.78, *p* < .0001), shorter duration (21.79 ± 26.01 vs 66.69 ± 67.67 and 24.56 ± 10.78, *p* < .0001), and more frequent dysphagia (35.3% vs. 3.4 and 6.3%, *p* = .001) compared with LGMD 2B and LSM patients. The prevalence of upper limb weakness (58.8% vs. 43.8% vs. 24.1%, *p* = .007), proximal dominance (86.8 and 68.8% vs. 27.6%, *p* < .0001), neck weakness (23.5 and 43.8% vs. 3.4%, *p* = .005), and severe muscle weakness (42.6 and 31.3% vs. 13.8%, *p* = .022) were higher in IMNM and LSM than in LGMD 2B. The highest peak CK value was observed for LGMD 2B [LGMD 2B vs IMNM and LSM: 7036 (3098, 9866) vs 6144 (3078,10,177) and 1444 (665,2980), *p* = .001]. LSM patients had a higher level of lactic dehydrogenase (LDH) [LSM vs IMNM and LGMD 2B: 808 (341, 1248) vs 569 (247,836) and 343 (280,455), *p* = .003] among three groups. In addition, the prevalence of anti-nuclear antibody (> 1:40) was higher than that in IMNM compared with LGMD 2B and LSM (58.2% vs. 0 and 6.25%, *p* = .0001). Additionally, LGMD2B patients showed more fat replacement (44.4% vs 16.9% and 0, *p* < .0001) on muscle MRI than IMNM and LSM patients (Table 3).

**Table 3:** Comparison of clinical and laboratory characteristics of IMNM and non-IIMs

Characteristics	IMNM(*n* = 68)	LGMD 2B(*n* = 29)	LSM(*n* = 16)	*P*
Female	45 (66.2)	22 (75.9)	7 (43.8)	.093
Age of onset	41.57 ± 14.45	21.66 ± 7.86	24.56 ± 10.78	<.0001^*^
Late onset (≥40)	35 (51.5)	0	1 (6.3)	<.0001^*^
Duration (months)	21.79 ± 26.01	66.69 ± 67.67	48.94 ± 79.07	<.0001^*^
Muscle weakness	62 (91.2)	27 (93.1)	15 (93.8)	.915
Lower limb weakness	48 (70.6)	17 (58.6)	11 (68.8)	.511
Upper limb weakness	40 (58.8)	7 (24.1)	7 (43.8)	.007
Lower limb dominant	52 (76.5)	24 (82.8)	16 (100)	.091
Proximal involvement	49 (72.1)	15 (51.7)	12 (75)	.115
Distant involvement	39 (57.4)	13 (44.8)	6 (37.5)	.259
Proximal dominant	59 (86.8)	8 (27.6)	11 (68.8)	<.0001^†^
Severe muscle weakness	29 (42.6)	4 (13.8)	5 (31.3)	.022^†^
Asymmetric	6 (8.8)	10 (34.5)	0	.003^†^
Neck involvement	16 (23.5)	1 (3.4)	7 (43.8)	.005^†^
MMT8	59.97 ± 15.80	70.86 ± 14.51	67.31 ± 12.35	.001^**^
Dysphagia	24 (35.3)	1 (3.4)	1 (6.3)	.001^*^
Muscular atrophy	10 (14.7)	8 (27.6)	2 (12.5)	.264
Myalgia	2 (33.8)	7 (24.1)	10 (62.5)	.058
ALT (0-40 U/L)	117 (64,241)	90 (64,157)	74 (46,150)	.348
AST (0-40 U/L)	85 (42,153)	68 (43,95)	62 (36,209)	.799
LDH (100-250 IU/L)	569 (347,836)	343 (280,455)	808 (341,1248)	.003^‡^
CK (26-200 IU/L)	2289 (894,5505)	4383 (1557,6485)	857 (325,1618)	.001^#^
Peak CK (26-200 IU/L)	6144 (3078,10,177)	7036 (3098,9866)	1444 (665,2980)	.004^#^
ANA (> 1:40)	40/66 (60.6)	0	1 (6.25)	.0001^*^
Muscle MRI	*N* = 59	*N* = 27	*N* = 16	
Inflammatory oedema	56 (94.9)	16 (59.3)	12 (75)	<.0001^*^
Fatty replacement	10 (16.9)	12 (44.4)	0	NA
Muscular atrophy	5 (8.4)	6 (22.2)	2 (12.5)	.207
Fascitis	4 (6.8)	0 (0)	0	NA

*IMNM* Immune-mediated necrotising myopathy, *LGMD* Limb-girdle muscular dystrophy, *LSM* Lipid storage myopathy, *ALT* Alanine aminotransferase, *AST* Aspartate aminotransferase, *LDH* Lactic dehydrogenase, *CK* Creatine kinase, *ANA* Anti-nuclear antibodies, *NA* not applicable

^*^ Bonferroni *P* < .017 between IMNM and LGMD2B or LSM; ^†^ Bonferroni *P* < .017 between LGMD 2B and IMNM or LSM; ^**^ Bonferroni *P* < .017 between IMNM and LGMD2B; ^‡^ Bonferroni *P* < .017 between LSM and IMNM or LGMD 2B,^#^ Bonferroni *P* < .017 between IMNM, LGMD 2B, and LSM pairwise

### Pathological characteristics of IMNM compared with non-IIM

**Fig. 1:**
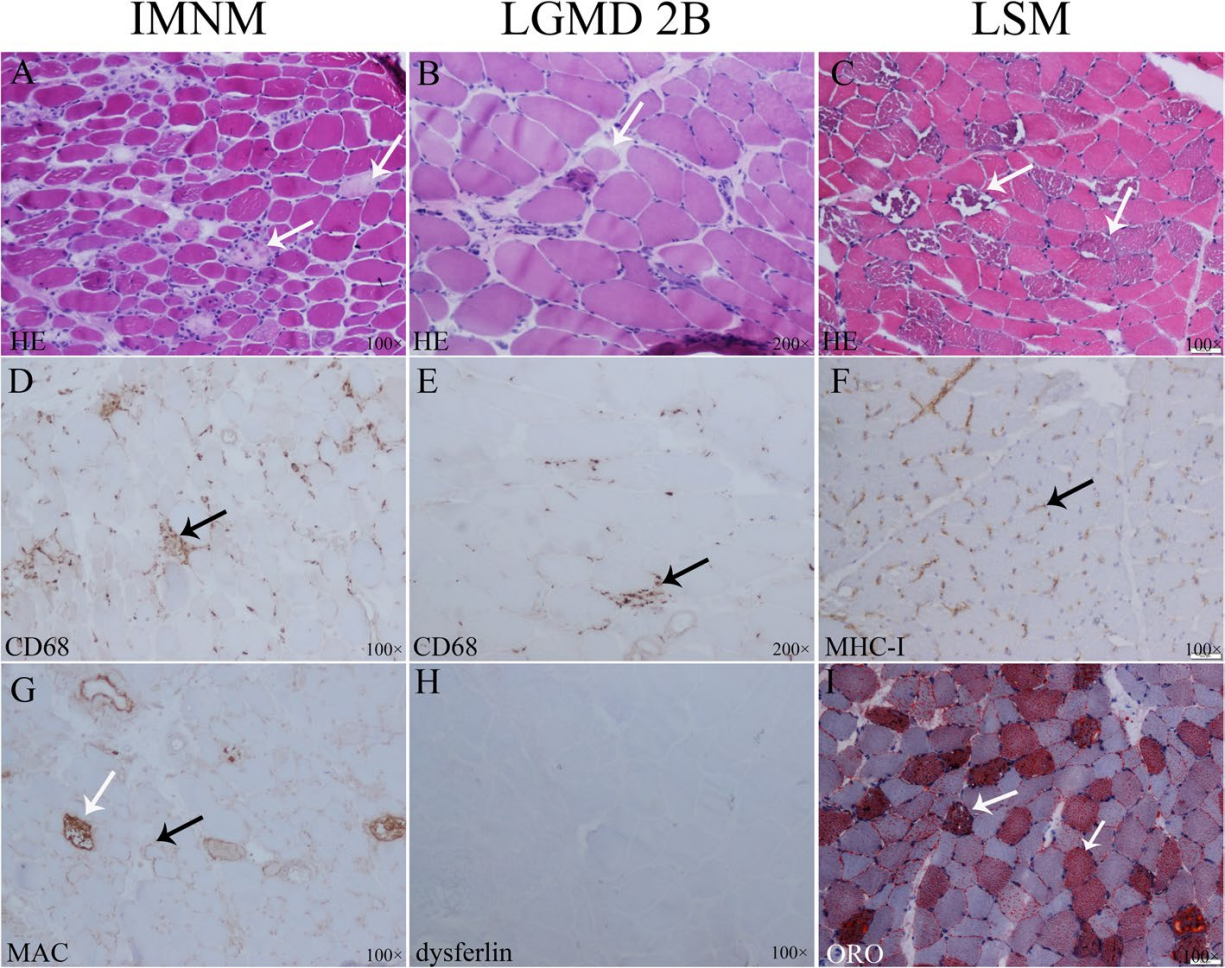
Pathological features of IMNM (A, D, G): A. scattered necrotic muscle fibres (white arrow); D. CD68^+^ macrophages expression on endomysia (black arrow); G. MAC deposition on sarcolemma of non-necrotic myofibres (black arrow) and sarcoplasm of necrotic muscle fibre (white arrow). Pathological features of LGMD 2B (B, E, H): **B**. muscle fibres of varying sizes (white arrow); E. CD68^+^ macrophages expression (black arrow); H. dysferlin expression deficient on sarcolemma. Pathological features of LGMD 2B (C, F, I): **C**. vacuolar muscle fibres (white arrow); F. non-overexpression of on MHC-I sarcolemma (black arrow); I. lipid droplet deposition in vacuolar muscle fibres (white arrow). A-C: HE staining; **D**-**H**: immunohistochemical staining; I. oil red O staining. IMNM, immune-mediated necrotising myopathy; LGMD, limb-girdle muscular dystrophy; LSM, lipid storage myopathy

The main pathological features of IMNM muscle were fibre necrosis (95.6%), MHC-I overexpression on the sarcolemma (83.8%), and CD4^+^ T cell infiltration (89.7%). However, muscle fibre necrosis was also observed in LGMD 2B (72.4%) and LSM (56.3%) patients. IMNM patients showed more severe necrosis (54.4% vs 13.8 and 12.5%, *p* < .0001), MHC-I overexpression on the sarcolemma (83.8% vs 37.9 and 12.5%, *p* < .0001) and CD4^+^ T cell perimysial expression (30.9% vs 7.1 and 12.5%, *p* < .0001) than LGMD 2B and LSM patients. MHC-I expression also occurred in 37.9% of LGMD 2B patients and in 12.5% of LSM patients, although diffuse MHC-I expression was only observed in IMNM (23.5%) patients. More connective tissue proliferation in muscle biopsy was observed in IMNM and LGMD than in LSM (44.1 and 37.9% vs 0, *p* = .005). MAC deposition was not a specific pathological performance of IMNM, which also occurred in 64% of LGMD 2B patients. However, only 20% of LSM patients present with MAC deposition. Dysferlin expression negative and lipid droplets in muscle fibres (especially in type 1 fibres) in ORO staining were the specific pathological features of LGMD 2B (92%) and LSM patients (86.7%), respectively (Table 4 and Fig. 1).

**Table 4:** Comparison of pathological characteristics of IMNM and non-IIMs

Characteristics	IMNM (*n* = 68)	LGMD 2B(*n* = 29)	LSM (*n* = 16)	*P*
Muscle fibre necrosis	65 (95.6)	21 (72.4)	9 (56.3)	<.0001^*^
Mild necrosis	28 (41.2)	17 (58.6)	7 (43.8)	
Severe necrosis	37 (54.4)	4 (13.8)	2 (12.5)	<.0001^*^
Connective tissue proliferation	30 (44.1)	11 (37.9)	0	.005^†^
MHC-I expression on sarcolemma	57 (83.8)	11 (37.9)	2 (12.5)	<.0001^*^
Focal expression	37 (54.4)	11 (37.9)	2 (12.5)	
Diffuse expression	16 (23.5)	0	0	<.0001^*^
CD4^+^ T cell	61 (89.7)	15/28 (53.6)	8 (50)	<.0001^*^
Endomysia	41 (60.3)	15/28 (53.6)	7 (43.8)	.461
Perimysium	21 (30.9)	2/28 (7.1)	2 (12.5)	.024^*^
CD8^+^ T cell	45 (66.2)	14/28 (48.3)	5 (31.3)	.027
Endomysia	29 (42.6)	14/28 (50)	5 (31.3)	.481
Perimysium	12 (17.6)	2/28 (7.1)	1 (6.3)	.258
CD68^+^ macrophage	50/66 (75.8)	14/25 (56)	10/15 (66.7)	.179
Endomysia	39/66 (59.1)	14/25 (56)	10 (66.7)	.798
Perimysium	17/66 (25.8)	2/25 (8)	1/15 (6.7)	.066
MAC	46/66 (69.7)	16/25 (64)	3/15 (20)	.002^†^
Sarcolemma of non-necrotic muscle fibre	34/66 (51.5)	15/25 (60)	1/15 (6.7)	.002^†^
Capillaries	26/66 (39.4)	5/25 (20)	2/15 (14.3)	.068

*IMNM* Immune-mediated necrotising myopathy, *LGMD* Limb-girdle muscular dystrophy, *LSM* Lipid storage myopathy, *MHC* Major histocompatibility complex, *MAC* Membrane attack complex

^*^P < .017 between IMNM and LGMD 2B or LSM; ^†^ *P* < .017 between LSM and IMNM or LGMD 2B

## Discussion

In our muscle biopsy cohort, 61.7% of patients had IIM. In the IIM group, DM was relatively easy to distinguish from IMNM by the presence of typical rashes (heliotrope sign and Gotrron sign) and DM-specific MSA. In addition, 44.1% of IMNM cases presented with severe muscle weakness, which is higher than that in DM, which can also differentiate IMNM from DM [18]. ASS patients were distinguished from other subgroups of IIM by the presence of anti-amino-tRNA-synthetase antibodies and extramacular manifestations (arthritis, Raynaud’s phenomena, mechanic’ hands, or lung involvement). In our muscle biopsy cohort, only three patients (0.6% in IIM) could be diagnosed with clinico-pathologically defined sIBM according to the criteria, which is much lower than that in the Caucasian cohort. We speculated that this may be associated with the missed diagnosis of sIBM due to insufficient understanding by clinicians and pathologists in the past. In addition, the incidence of sIBM in different ethnic groups may be distinct, which may also contribute to the lower frequency of sIBM in our cohort.

LGMD 2B and LSM were the most common non-IIM that shared similar manifestations with IMNM in our cohort [25], in line with the high prevalence in the Chinese population [26]. However, IMNM has an older age of onset, while the other genetic myopathies have a younger onset age. In addition, the disease course of IMNM is shorter than that of hereditary myopathy. Demographic characteristics seem to vary according to the underlying aetiology. Middle-aged onset and subacute duration suggest IIM; however, young patients present with slowly progressive proximal muscle weakness that can be difficult to differentiate clinically from LGMD. Mohassel et al. [27] reported an anti-HMGCR-positive IMNM case with a more indolent disease course but favourable clinical response to immunotherapy, which is easy to confuse with muscular dystrophy. Tanboon et al. [28] also reported that concurrent anti-HMGCR antibodies and gene mutations indicated the possibility of co-occurrence of IMNM and muscular dystrophy. Thus, testing for these autoantibodies should be an essential part of the evaluation of children with symptoms resembling hereditary muscular disorders. In addition, upper limb weakness and dysphagia are more common in IMNM than in LGMD and LSM, although cervical flexor weakness is more common in LSM. Asymmetric muscle weakness is present only in LGMD 2B patients [29]. The above information reminds muscle specialists, neurologists, or rheumatologists about the necessity of a comprehensive and systemic examination of whole-body muscle strength for muscular diseases.

IMNM, LGMD 2B, and LSM had a significant elevation of CK in this study, although the highest level of peak CK appeared in LGMD 2B. A previous study reported that CK levels in IMNM are always up to 10–15 times the upper normal level, although in LGMD 2B, it can increase to more than 20 times. The study also shows that significant CK elevation indicates a higher probability of muscular dystrophy than IMNM [30]. The level of LDH in LSM patients can reach 808 (341, 1248) U/L, with the highest value of up to 2433 U/L, which is higher than that in the other groups. Zhang et al. also observed predominantly higher levels of LDH in LSM [31]. The reason for this is still unclear. LDH has isoforms of the liver and muscle. The abnormally high level of LDH in LSM patients may be due to the presence of lipid or glucose metabolic dysfunction and increased liver types. Therefore, identifying the isoforms may help determine their source and distinguish IMNM from metabolic myopathy.

Muscle fibre necrosis is not a specific manifestation of IMNM, which also occurs in patients without IIM. However, the proportion and degree of fibre muscle necrosis were significantly higher than in non-IIM, and diffuse expression of MHC-I and CD4^+^ T cell perimysial infiltration were more specific in IMNM. MAC deposition is less common in LSM, indicating that the complement pathway is less involved in the pathogenesis of LSM. Immunohistochemical staining of dysferlin, ORO, and PAS in patients with suspected muscular dystrophy and metabolic myopathy are helpful for clinicians to exclude IMNM from muscular dystrophy and metabolic myopathy [25, 32, 33]. LGMD 2B had a significantly longer disease course than IMNM and LSM, but no significant difference in the proportion of connective tissue hyperplasia was observed in muscle pathology among the three subgroups. However, patients with LGMD 2B had most fatty replacement and muscle atrophy in muscle MRI. These findings suggest that connective tissue hyperplasia in IMNM may appear in very early stages of the disease, while fat replacement and muscle atrophy caused by the long course of disease are easily observed with MRI.

This study had some limitations. This is a retrospective study. The diagnosis of the patients included in the study was based on the previous clinical diagnosis in the medical records. Some hereditary myopathies were diagnosed according to pathological findings and were not confirmed by genetic tests, or the pathological features were inconsistent with genetic tests. Such patients could not be re-classified into any defined myositis or myopathies in this study, which have led to a high proportion of undiagnosed patients in the study.

## Conclusion

This study investigated the distribution of various types of myopathies and analysed the characteristics of IMNM in a single-centre muscle biopsy cohort. It is still important for rheumatologists to distinguish IMNM from non-IIM and obtain an accurate diagnosis. To achieve this, detailed analysis of the clinical and pathological characteristics of IMNM is useful, especially the differences between IMNM and similar myopathies, such as PM and muscular dystrophy.

## Supplementary Information

**Additional file 1.**

## Data Availability

The data that support the findings of this study are available from the corresponding author but restrictions apply to the availability of these data, which were used under license for the current study, and so are not publicly available. Data are however available from the authors upon reasonable request and with permission of the China-Japan Friendship Hospital.

## Abbreviations

ACR: American College of Rheumatology
ANA: Anti-nuclear antibody
ASS: Anti-synthetase syndrome
CK: Creatine kinase
CTD: Connective tissue disease
DM: Dermatomyositis
ENMC: European Neuromuscular Centre
EULAR: European League Against Rheumatism
HMGCR: Anti-3-hydroxy-3-methylglutaryl-coenzyme A reductase
sIBM: Sporadic inclusion body myositis
IIM: Idiopathic inflammatory myopathies
IMNM: Immune-mediated necrotising myopathy
LDH: Lactic dehydrogenase
LGMD: Limb girdle muscular dystrophy
LSM: Lipid storage myopathy
MAA: Myositis-associated antibodies
MAC: Membrane attack complex
MHC: Major histocompatibility complex
MMT: Manual muscle testing
MSA: Myositis-specific antibody
NGS: Next-generation sequencing
ORO: Oil red O
PAS: Periodic acid-Schiff
PM: Polymyositis
SRP: Signal recognition particle
