# Supplementary material for: Clinical and pathological features of immune-mediated necrotising myopathies in a single-centre muscle biopsy cohort
Source: BMC Musculoskelet Disord. 2022 May 6;23:425. doi: 10.1186/s12891-022-05372-z (PMC9074315; doi:10.1186/s12891-022-05372-z)
Supplement: Supplementary file 1 — Additional file 1. [file 12891_2022_5372_MOESM1_ESM.doc]

Additional table 1 Diagnosis criteria of various muscular diseases in a muscle biopsy cohort

| Disease | Diagnostic criteria |
| --- | --- |
| Muscular Dystrophy | 1. Muscle weakness; 2. Muscular dystrophy-associated protein expression deficient on histochemical staining in pathological species; and/or 3. Identification of mutations in the muscular dystrophy associated genes by genetic testing. |
| Limb gridle muscular dystrophy 2B | 1. Muscle weakness/CK elevation; 2. Dysferlin expression deficient in muscles; and/or 3. Identification of dysferlin gene mutations [1]. |
| Other types of muscular dystrophy | 1. Muscle weakness/CK elevation; 2. Muscular dystrophy associated protein (e.g. Dystrophy, Sarcolemma expression deficient/decreased in pathological species; and/or 3. Identification of dystrophy gene mutations [1]. |
| Metabolic myopathy | Metabolic myopathies are a heterogeneous group of rare disorders leading to primarily skeletal muscle damage and frequent involvement of other organs, due to defects of enzyme activities regulating carbohydrates, lipids or mitochondrial metabolism[2]. |
| Lipid storage myopathy | 1. Muscle symptoms; 2. Lipid droplet accumulation in muscle fibres; and/or 3. Identification of LSM-associated gene (e.g. ETFA, ETFB, ETFDH, ABHD5, PNPLA2) mutations [3]. |
| Mitochondrial myopathy | 1. Muscle symptoms; 2. Suggestion of a large amount of mitochondrial accumulation in muscle fibres on Gomri staining; and/or 3. Identification of mitochondrial myopathy associated gene (e.g. mtDNA) mutations [4]. |
| Glycogen storage myopathy | 1. Muscle symptoms; 2. Abundant accumulation of glycogen in muscle species; and/or 3. Identification of glycogen storage disease associated gene (e.g. GBE1, RBCK1, GYG1) mutations [5]. |
| Endocrine myopathy | 1. Muscle symptoms/CK elevation; 2. Exclusion of other muscular diseases; 3. Hypothyroidism; 4. weakness improves with anti-hypothyroid treatment. |
| Neurogenic myopathy | 1. Muscle symptoms/CK elevation; 2. With neurogenic changes, such as neurogenic changes in electromyogram; 3. Exclusion of other muscular diseases; |
| Other CTD accompanied with skeletal muscle symptoms | 1. Definite diagnosis of CTDs (i.e., rheumatoid arthritis, systemic lupus erythematosus); 2. With muscle symptoms (ie, muscle weakness, myalgia); 3. Exclusion of any type of myositis (according to 2017 EULAR/ACR criteria). |
| Myopathy induced by external factors | 1. Muscle symptoms/CK elevation; 2. Exclusion of other muscular diseases; 3. With clear incentives including infection, exercise and drugs. |
| Asymptomatic hyperCKemia | 1. CK elevation without muscle symptoms; 2. Exclusion of other muscular diseases. |

CTD, connective tissue diseases; CK, creatine kinase

[1] Mercuri E, Bönnemann CG, Muntoni F. Muscular dystrophies. Lancet 2019;394:2025–38. https://doi.org/10.1016/S0140-6736(19)32910-1.

[2] Antonio Toscano, Emanuele Barca, and Olimpia Musumeci. Update on diagnostics of metabolic myopathies. Curr Opin Neurol 2017, 30: 0(5):553-62. https://doi.org/10.1097/WCO.0000000000000483

[3] Liang WC, Nishino I. Lipid storage myopathy. Curr Neurol Neurosci Rep 2011;11:97–103. https://doi.org/10.1007/s11910-010-0154-y.

[4] Ahmed ST, Craven L, Russell OM, Turnbull DM, Vincent AE. Diagnosis and Treatment of Mitochondrial Myopathies. Neurotherapeutics 2018;15:943–53. https://doi.org/10.1007/s13311-018-00674-4.

[5] Kanungo S, Wells K, Tribett T, El-Gharbawy A. Glycogen metabolism and glycogen storage disorders. Ann Transl Med 2018;6:474. https://doi.org/10.21037/atm.2018.10.59.
